# Ligand Binding and Signaling of HARE/Stabilin-2

**DOI:** 10.3390/biom9070273

**Published:** 2019-07-11

**Authors:** Edward N. Harris, Fatima Cabral

**Affiliations:** Department of Biochemistry, University of Nebraska, Lincoln, NE 68588, USA

**Keywords:** stabilin, HARE, receptor-mediated endocytosis, scavenger receptor, hyaluronan

## Abstract

The Stabilin receptors are a two-member family in the type H class of scavenger receptors. These dynamic receptors bind and internalize multiple ligands from the cell surface for the purpose of clearing extracellular material including some synthetic drugs and for sensing the external environment of the cell. Stabilin-1 was the first receptor to be cloned, though the biological activity of Hyaluronic Acid Receptor for Endocytosis (HARE)/Stabilin-2 was observed about 10 years prior to the cloning of Stabilin-1. Stabilin-1 has a more diverse expression profile among the tissues than HARE/Stabilin-2. This review will focus on HARE/Stabilin-2 and its interactions with hyaluronan, heparin, and phosphorothioate antisense oligonucleotides and what is known about how this receptor participates in signaling upon ligand binding.

## 1. Initial Discovery

**Figure 1:**
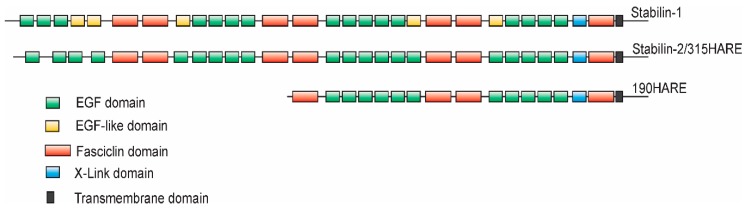
**Topology of Stabilin-1 and Stabilin-2.** The overall topologies of human Stabilin-1 and human Stabilin-2, including the 190HARE, are very similar with an overall protein sequence identity of 39% and similarity of 56% between the two large receptors. The topology algorithm was derived using the SMART program and the homology was calculated using BLAST program of the National Center for Biotechnology Information.

**Figure 2:**
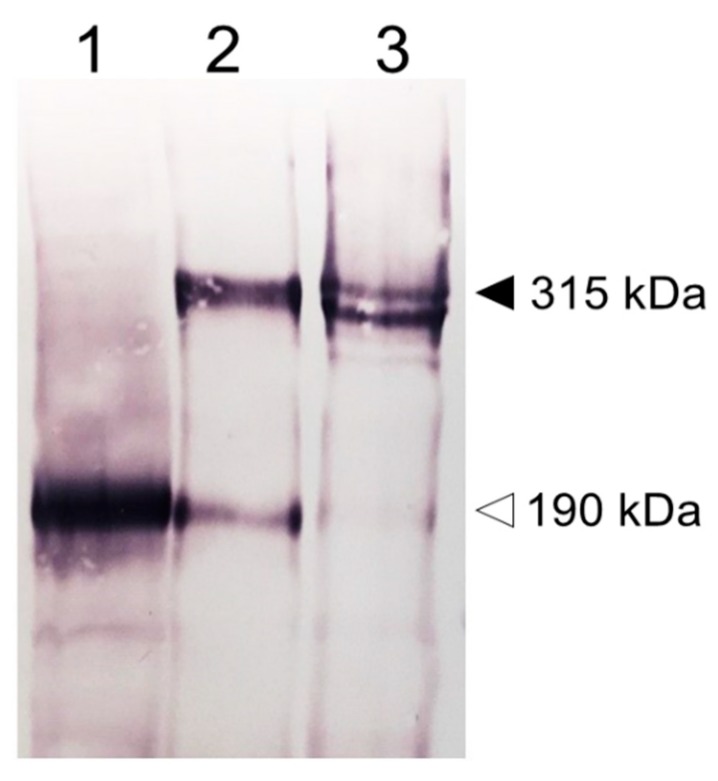
**Protein characterization by SDS-PAGE**. Stable cell lines expressing human (**1**) 190HARE, (**2**) Stabilin-2 or 315HARE, and (**3**) Stabilin-1 were prepared as whole cell lysates and 20 μg of lysates were separated by 5% denaturing gel electrophoresis without reduction. The dark arrowhead (315 kDa) indicates the monomer of Stabilin-2 and doublet of Stabilin-1 and the open arrowhead (190 kDa) indicates the presence of the 190HARE in cell lysates expressing a construct that only codes for the 190HARE and Stabilin-2. All plasmid constructs (pcDNA5/FRT/V5-6xHis, Invitrogen, Carlsbad, CA) used to express these proteins contained the cytomegalovirus promoter for protein expression and C-terminal tags, 6xHis and V5. Anti-V5 antibody was used to detect the recombinant protein followed by a secondary antibody for standard colorimetric detection with alkaline phosphatase.

The discovery of the cloned Stabilin receptors began with Stabilin-1 in the early 1990s. A monoclonal antibody raised against whole human spleen homogenate produced a monoclonal antibody (MS-1) against an antigen that was initially identified as a large protein that is preferentially expressed in the sinusoidal endothelia of spleen, lymph node, liver, and adrenal cortex [1]. The identified MS-1 antigen is an inducible protein not found in normal skin lesions, but rather in diseased skin and some cancers such as psoriasis, melanoma, and T-cell lymphomas. Induction of MS-1 antigen is stimulated by Interleukin (IL)-4 and Dexamethasone and inhibited by tumor necrosis factor (TNF)-α, IL-1β, and IL-6 [2] in cultured human peripheral blood monocytes [3,4]. The use of the MS-1 antibody allowed for immunoprecipitation of the MS-1 antigen which was subjected to matrix-assisted laser desorption ionization—time of flight (MALDI-TOF) analysis for protein identification. The same authors used hyaluronic acid (HA) affinity chromatography to identify a similar protein that bound HA with a high degree of topology between both receptors [3]. However, several years prior to the publication of Politz et al. [3], the rat HA-binding receptor was discovered from rat liver sinusoidal endothelial cells (SEC HA receptor) [5,6] and identified as the primary HA turnover receptor named HARE (Hyaluronic Acid Receptor for Endocytosis) [7]. Due to their topological similarity, the receptors that were formerly named MS-1 antigen or protein, FEEL-1, CLEVER-1 and FEEL-2, HARE are now almost universally recognized as Stabilin-1 and Stabilin-2, respectively [3,8,9] (Figure 1 as annotated by [10,11]). Although there was a bit of confusion as to the number of protein subunits composing these receptors during their initial characterization [1,7,12], cloning and expression in stable cell lines have confirmed that Stabilin-1 is a monomer with two forms expressed as a tight doublet at about 320 kDa by sodium dodecyl sulfate—polyacrylamide gel electrophoresis (SDS-PAGE) [13] and a possible smaller isoform at around 140 kDa by SDS-PAGE which has not been confirmed by other sources [14]. Human Stabilin-2 is expressed as two monomers or isoforms at 315-kDa and 190-kDa by SDS-PAGE [15] (Figure 2) which is similar to rat Stabilin-2 expressed as 300-kDa and 175-kDa isoforms [7]. Both human and rat receptors have nearly the same number of amino acids but the higher mass of the human receptor is solely contributed by post-translational glycosylation of the receptor. In native SECs, the ratio of the large to small isoforms is close to a 1:1 ratio in liver, whereas in the spleen, it is 2:1 or 3:1 [7]. However, in stable cell lines, the isoform ratio is closer to 8–10:1, suggesting that the mechanism for the appearance of the 190HARE is more robust in primary cells [15]. It is thought that these isoforms are a product of proteolytic cleavage that occurs somewhere between the trans-Golgi network and the plasma membrane during synthesis. Using [35S] labeled tracking, the appearance of the small isoform appeared immediately after the large isoform was fully glycosylated (unpublished data). For simplicity in nomenclature, the large isoform of Stabilin-2 will be referred to as Stabilin-2 and the smaller isoform of the same receptor will be referred to as the 190HARE.

## 2. Tissue Expression

The location of expression determines functionality, especially with receptors that bind to multiple ligands. One of the first studies for rat 175HARE expression was performed by Zhou et al. in which crude tissue extracts were produced in Nonidet NP-40 from spleen, lung, small intestine, large intestine, muscle, kidney, heart, brain, and liver. The protein was immunopurified by a monoclonal antibody bound to sepharose beads against rat Stabilin-2 and then separated on a 5% acrylamide gel for SDS-PAGE and probed with mAb-235 against rat 175HARE and radiolabeled HA. These results indicated that Stabilin-2 was only expressed in liver and spleen tissues and the ratio of the isoforms varied between these two tissues [7]. Later work published from the same laboratory confirmed by histological staining that the sinusoids of liver, medullary sinuses of lymph node, and venous sinuses of spleen were enriched in Stabilin-2 expression [16].

One year after the initial publication, Politz et al. used Northern blots to confirm the expression of both human Stabilin-1 and Stabilin-2. This expanded the known tissues for these two receptors where Stabilin-2 expression was strong and verified in liver, spleen, lymph node with much lighter expression in heart, placenta, skeletal muscle, pancreas, thymus, and bone marrow [3]. Stabilin-1 had strong expression in placenta, liver, spleen, and lymph node with lighter expression in peripheral blood leukocytes [3]. A year after this publication, Falkowski et al. took a closer look using immunohistochemistry using an antibody raised against a Stabilin-2 peptide and detected mouse Stabilin-2 expression in tissues that were negative for expression in previous work. Expression was specifically in the ocular corneal epithelium, ocular cuboidal epithelium, cardiac mensenchyme, brain ependymal/cuboidal epithelial cells lining the ventricles, and the epithelial cells of the renal papillae [17]. It is understandable that these were missed previously as these tissues have only a single cell layer expressing the receptor that would easily get diluted out in a whole-tissue homogenate. Recently, Stabilin-2 expression was found in myoblasts and contributes, in part, to their fusion during differentiation or regeneration [18]. Current online genomic databases such as http://gtexportal.org/home are also helpful in determining Stabilin expression throughout many tissues, but results should be interpreted cautiously as transcriptomic data is fully correlative for protein expression [19,20].

## 3. Hyaluronan Binding and Endocytosis

Hyaluronan (HA) is a very long polymer containing disaccharides composed of glucuronic acid β1-3 linked to *N*-acetylglucosamine and these disaccharides are linked by β1-4 glycosidic bonds. HA is a necessary extracellular matrix molecule essential for a wide variety of functions including joint health and lubrication, ocular integrity, skin hydration, fertility, cancer metastasis, and many more physiological functions [21,22,23,24,25]. Likewise, turnover of HA is very dynamic in which hyaluronidases/spreading factor/mechanical forces/oxidative stress break down very large HA in the tissues which then drains into the lymphatics and finally into the blood [26]. It is the function of clearance receptors in the lymphatics and circulatory systems to maintain low levels of HA in these fluids. Early studies of HA metabolism reveal that [3H]Hyaluronan injected via the marginal ear vein of a mature rabbit is rapidly cleared in about 20 min from plasma with a half-life of 3.5–6 min [27]. Tissue accumulation of [3H]Hyaluronan was primarily in liver, spleen, lymph nodes, and bone marrow, all tissues containing high expression levels of Stabilin-2 with large overlaps in Stabilin-1 expression [28]. Furthermore, the liver SECs, and not the Kupffer or hepatocytes, have the highest accumulation of hyaluronan [29]. Both Stabilin-1 and Stabilin-2 are highly expressed in liver SECs among other tissues previously mentioned. Although both Stabilins contain X-Link domains, the Stabilin-1 domain is inactive for hyaluronan binding [3], thus Stabilin-2 is the primary scavenger receptor for systemic HA. 

**Figure 3:**
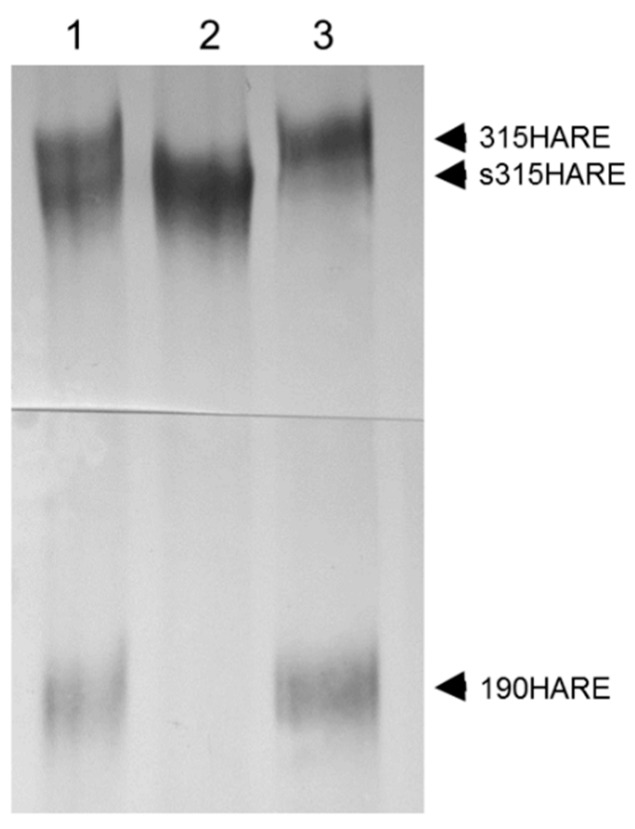
**Membrane attachment is required for production of 190HARE.** Cells expressing either 315HARE (Stabilin-2) or secreted 315HARE (lacking the transmembrane and cytoplasmic domains) were lysed in conditioned medium and immunoprecipitated with Ab30 conjugated to sepharose beads. Immunoprecipitated proteins from (**1**) cell lysate from both 315HARE and s315HARE were mixed 1:1, (**2**) s315HARE lyste, and (**3**) 315HARE lysate were separated by 5% SDS-PAGE on a 20 cm gel, blotted, and probed with anti-V5 antibody for protein detection.

To investigate the HA–Stabilin-2 interaction more closely and in detail, we expressed the human 190HARE (smaller isoform) in Flp-In HEK 293 cells [30]. Using a variety of techniques including HA degradation monitoring, Scatchard analysis, and internalization vs binding assays, the disassociation rate (kD) for the 190HARE was calculated to be 7.2 nM which was similar to the kD in recombinant SK-Hep-1 cells expressing the rat 175HARE at 4.1 nM [31]. In addition, the 190HARE recycling time on the cell surface was 7–9 min which was also similar to what had been calculated for recycling time for native rat Stabilin-2 receptors in primary liver SECs [32]. These physical properties revealed that the recombinant 190HARE was a good model for the native Stabilin-2 receptors in SECs, a cell type that is difficult to culture and maintain its differentiation for more than two days. Full-length Stabilin-2 was also expressed in stable cell lines under the same promoter and cell type as the 190HARE. For reasons unknown, the expression level of Stabilin-2 was always robust, but significantly lower than the 190HARE. In addition, the 190HARE was always co-expressed with Stabilin-2 in these cell lines, suggesting that production of the small isoform is a natural byproduct of the full-length receptor. Endocytosis of HA in the Stabilin-2 cells was not significantly different from the 190HARE when accounting for the number of cells and receptor expression levels. Expressing both isoforms as soluble ecto-domains and purifying them by nickel chelate chromatography revealed that the binding of HA in an ELISA-like format for both receptors was nearly equivalent (20.7 ± 6.4 nM for 190-HARE, 9.9 ± 1.2 nM for Stabilin-2) [15], further demonstrating that the binding occurs primarily within the X-Link domain and not in other areas of the receptor. X-Link binding of HA was further confirmed in cells expressing a receptor without the X-Link domain as these cells were not able to internalize any [125]I-HA [33]. Curiously, expression of the Stabilin-2 ecto-domain did not result in producing a small isoform, indicating that membrane attachment is required for small isoform (190HARE) expression (Figure 3).

The X-Link domain of Stabilin-2 has the highest homology to another well-known hyalectin, tumor necrosis factor inducible gene 6 protein (TSG-6), a protein whose expression is induced by inflammatory mediators, growth factors, ovulation inducers, and matrix remodeling [34,35,36]. In silico modeling based on the structural work performed with TSG-6 reveals that HA binds within a groove of the X-Link domain and interacts directly with tyrosine residues through hydrogen bonding [37]. This TSG-6 model has been upgraded more recently to reflect the more intimate interaction of HA with the X-Link domain in which binding is mediated with a combination of ring stacking interactions, hydrogen bonds, salt bridges, and hydrophobic interactions along the binding groove [38]. The similarity of HA binding with TSG-6 and the 190HARE was further corroborated by another independent study using atomic force microscopy (AFM) to determine the bond strength between HA and several hyalectins. Using biotinylated HA at the tip of the AFM probe and proteins bound to solid substratum, the mean rupture force could be calculated between a single polymer of HA and the hyalectin. Both TSG-6 and 190HARE had a mean rupture force with HA at 24 and 25 picoNewtons (pN), respectively, further suggesting that both proteins interact with HA in a similar fashion. Comparatively, HA rupture forces were 34 pN for CD44, 37 pN for versican, and more than 52 pN for aggrecan X-Link domains [39]. Although the binding affinity of HA for 190HARE is in the low nanomolar range, the bond strength with which it binds is one of the weakest among the hyalectin family. Biologically, this is logical as this is a constitutively recycling receptor that must bind HA with high specificity and then release this cargo in the endosomes. The other hyalectins with higher rupture forces are all HA-binding proteins for structural purposes, hence the more stable interactions.

Data derived from cell models suggests that the degradation of internalized HA does occur, though this is highly dependent on the cell type. For example, SK-Hep-1 cells are of endothelial origin [40], but are very active in transcytosis. It is estimated that about half of the HA that is internalized by SK-Hep-1 cells stably expressing the rat 175HARE is delivered back to the extracellular space entirely intact [41]. In 190HARE cells (HEK293 background), most of the HA is degraded and the ratio of intact versus degraded HA may be measured by cetylpryridinium chloride precipitation [41,42]. Primary SECs have a much greater capacity for endocytosis and degradation of HA than these stable recombinant cell models. HA is internalized by Stabilin-2/HARE and transported to lysosomes which contain lysosomal β-glucuronidases, β-*N*-acetylglucoaminidase, and hyaluronidases for polymer degradation. The HA polymer is degraded into monosaccharides that are transferred to the cytosol where glucuronic acid is converted by isomerases for metabolism. The *N*-acetylglucosamine is also transferred to the cytoplasm and phosphorylated then deacetylated for the eventual formation of acetate, lactate, and fructose [42,43,44].

## 4. Heparin Clearance by Stabilin-1 and Stabilin-2 

Heparin is a complex polysaccharide made up of two base sugars, *N*-acetylglucosamine and glucuronic (epimer: iduronic) acid. These two sugars may be modified in the Golgi of the cell to give rise to up to 32 different disaccharide combinations resulting in more than 100 trillion unique heparin polymers [45]. The heparin core protein is Serglycin which is enriched in serine and glycine residues in which the serine is modified with the heparin polymer. As a post-translational product of Serglycin, the first modifications occur in the ER and a step-wise process of elongation and modification to the sugar chain occurs in the Golgi [46]. Heparin is produced in mast cells of the immune system which are enriched in the gut mucosa of mammals [47]. Heparin was discovered in 1916 by a medical student, Jay McLean, working in the laboratory of Professor Howell, and its use as an anticoagulant in medical applications began in the mid-1930s [48]. Three forms of heparin for use in medicine are: purified unfractionated heparin (UFH) with a mass average of 13.5 kDa, the processed UFH in the development of low-molecular-weight heparin (LMWH) with a mass average of 3.5 kDa, and the synthetic pentasaccharide, Fondaparinux [49]. 

Regardless of the route of administration, heparin clearance from circulation is dependent on the liver and kidneys. Longer polymers tend to be taken up by the liver for degradation and then undergo filtration by the kidney. It was discovered that the SECs of the liver are the primary site for heparin clearance from blood [50,51]. The primary, though not only, receptor for heparin clearance was discovered to be Stabilin-2 using direct binding methods of purified protein with radioactively labeled heparin [52]. Heparin has a high electronegative charge and, thus, it is a “sticky” molecule that binds to many components on the surface of cells. Cells that do not express Stabilin-2 will slowly internalize heparin during the process of normal membrane turnover. However, cells that do express either Stabilin-2 or 190HARE take up much more heparin compared to the same type of cells that do not express these receptors. Both UFH and LMWH are taken up by Stabilin-2 [49] and Stabilin-1 is also involved with heparin binding and endocytosis [53]. 

As previously mentioned, heparin is a very heterogeneous molecule with respect to both size and modification. Currently, therapeutic heparin is a natural product that comes from swine gut mucosa from which it is purified and refined [54,55]. The goal of making synthetic heparin has been around for decades [56,57] and is currently becoming a reality with the development and refinement of chemoenzymatic processes [58,59,60,61,62]. Using homogenous synthetic heparin polymers, we have found that the heparin polymer needs to be sulfated for binding with Stabilin-1 or Stabilin-2. The nonsulfated polymer, heparosan, does not interact with the Stabilin clearance receptors and may be a good candidate as a vehicle for drug delivery [63,64]. The minimal binding requirement is for the polymer to be at least 10 sugars long and the presence of the 3-*O* sulfate increases binding affinity at least fivefold [53]. To determine how important the 3-*O* sulfate is for overall metabolism and uptake in the liver, we subcutaneously injected mice with physiological doses of dodecamers (12mers) with or without a single 3-*O* sulfation residue (15 vs. 14 total sulfate groups along a single dodecamer). The 3-*O* sulfate routed the polymers through the liver for endocytosis and degradation and also increased the half-life of the 12mer in blood. Without the 3-*O* sulfation, the polymer had a far higher chance of getting immediately filtered by the kidneys [65]. From these experiments, we deducted that polymer charge is not the only defining characteristic of heparin–Stabilin interactions. The orientation of the charge is just as important as the charge itself for optimal binding with the receptor. The single 3-*O* modification along the polymer affected affinity to Stabilin-2 and liver, increased the half-life in blood, and, possibly, allowed interaction of heparin with other blood components. It is clear that the Stabilin-2 receptor directly affects the half-life of systemic UFH and LMWH. 

The precise location of where heparin binds with the Stabilin-2 receptor is not known and the primary sequence of the amino acids does not reveal an obvious cluster of positively charged amino acids that are common for many heparin binding proteins. Since both Stabilin-2 and 190HARE bind with heparin with the same affinity [52], the putative binding site(s) are only in the 190HARE sequence and the N-terminal half of Stabilin-2 does not contribute to binding, despite similar domain organization (Figure 1). Stabilin-2 (and Stabilin-1) are both extensively folded and it is hypothesized that a heparin binding domain/groove is formed as distal amino acids are brought together in close proximity to form this site. Unfolding of the receptor through reduction of the disulfide bonds renders the receptor unable to bind any ligands. 

Heparin does not interfere with HA binding on Stabilin-2. In the TSG-6 model, the X-Link domain may bind either HA or heparin, but not both together at two different binding sites within the module [66]. In contrast, heparin binds to a distal site on Stabilin-2 and not within the X-Link domain. Thus, both HA and heparin may bind with Stabilin-2 simultaneously [67].

## 5. Stabilin Interactions with Antisense Oligonucleotides (ASOs) 

**Figure 4:**
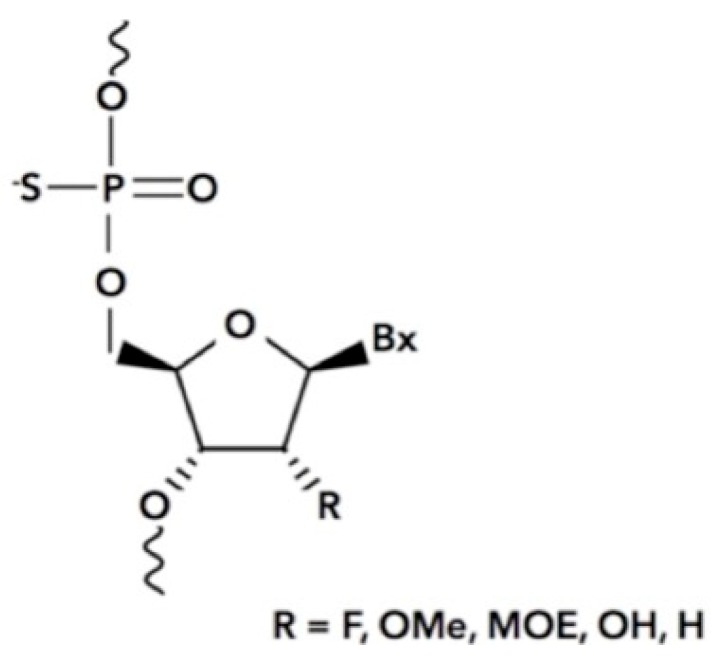
**The phosphorothioate antisense oligonucleotide**. The nonbridging oxygen of the phosphodiester is substituted with a sulfur atom to produce the phosphorothioate which is required for efficient Stabilin binding. Bx = purine/pyrimidine base, R = variable chemistries of both natural and unnatural modifications which includes fluoro (F), O-methyl (OMe), methoxyethyl (MOE), and hydroxyl (OH).

Antisense oligonucleotides are chemically synthesized oligonucleotides that bind RNA through Watson–Crick base pairing and mediate its destruction via endogenous enzymes such as Dicer and RNase H [68,69]. These therapeutic drugs are specific for their targets owing to the nucleotide sequence and have relatively few side effects. ASOs are resistant to degradation due to the altered synthetic chemistry of the backbone in which the phosphodiester is substituted for a phosphorothioate (PS) or phosphorodiamidate chemistry which is resistant to nucleases (Figure 4). In addition, the 2′ position of the ribose may be modified with any number of chemistries such as a methoxyethyl (MOE), fluro, O-methyl (OMe), and other chemistries to enhance stability and facilitate binding to target RNAs [70]. The chemistry for specificity and stability is well known, however, delivery to the correct tissues has been challenging [70]. Many advancements in the past five years have begun to solve this issue by conjugating ASOs with targeting ligands such as *N*-acetylgalactosamine for targeting the liver parenchyma [71] and the GLP1 peptide for targeting the pancreas [72]. Other delivery methods include encapsulating the ASO with lipid-based molecules to form a nanoparticle comprising phosphatidylcholine, cholesterol, polyethylene glycols, and amino lipids for delivery by blood infusion. The use of naked or gymnotic delivery methods utilizes the backbone chemistry for subcutaneous and intrathecal administration in which the phosphorothioate chemistry is the most popular as it sticks to cellular surface membranes and proteins for eventual internalization [73].

Only a few receptors are known to internalize phosphorothioate-based (PS) ASOs and they are the asialoglyoprotein receptor (ASGPR) [74], scavenger receptor A (SR-A) [75], epidermal growth factor receptor (EGFR) [76], and the Stabilins [77]. Of this group, the Stabilins have the highest affinity, avidity, and turnover rate in comparison with the others, thus, the Stabilins are the primary clearance mechanism for the PS-ASOs due to these properties and their location of expression. Using a 5-10-5 MOE-flanked, single-stranded PS-ASO that was iodinated with [125I], it was found that there was no competition with HA binding in 315HARE cells. There was partial competition with heparin, however, and it is not known at this time whether that is due to competition for the same binding site on the receptor or at a secondary site which affects ligands at the primary binding site [77]. 

Since ASOs may be synthesized with a wide variety of chemistries, we asked which chemistries are the best ligands for the Stabilins. In Miller et al., we used an iodinated single-stranded 5-10-5 MOE fully PS gapmer as the probe and competed it with a 200-fold concentration of a variety of ASOs of different lengths and chemistries [77]. The noncompeted PS-ASO bound to the *secreted* ecto-domain of the 190HARE (s190HARE) in an enzyme-linked immunosorbent assay (ELISA)-like assay at 140 nM. The results show that as the number of flanking residues decrease in number, so does the competition with the 20mer ASO. Using a fluorescence polarization assay, we determined that a full PS oligo has the highest affinity for s190HARE which decreases as PO residues are substituted in. Modifications of the 2′ ribose has very little effect on Stabilin binding and the nucleotide sequence has no effect on binding. Single-stranded oligos have a much higher avidity for the Stabilins than double-stranded oligos. It is thought that the double-stranded oligo is more rigid and interactions between the oligo and the Stabilins need to be flexible to accommodate the interactions of the anionic sulfur atom with the protein surface [70,78]. Knowing that the avidity of the ligands may be modulated by manipulating the number and spacing of PS residues, it is possible to increase or decrease the half-life of the ASO in blood, thereby exposing other tissues for either longer or shorter times to the ASO. 

An independent genome-wide association study using a hybrid mouse diversity panel confirmed that Stabilin-2 and vesicle-associated membrane protein 3 (Vamp3) are closely associated with hepatic ASO deposition in more than 100 genetically unique strains of mice [79]. Throughout this work, it was confirmed that (i) mice strains with lower Stab2 expression contained significantly lower hepatic PS-ASO accumulation,(ii) immunohistochemistry of liver sections confirmed that the SECs of the liver were a site of high PS-ASO accumulation, and (iii) Stab2^-/-^ mice accumulated a significantly lower amount of ASOs in their liver and spleen. What was surprising in this work was that efficacy of ASO-mediated knockdown in the liver of WT and Stab2^-/-^ mice was similar, leading to the idea that initial uptake is front-loading the nonproductive pathway and that low amounts of ASOs are very effective at mRNA suppression. ASO accumulation was primarily in the SECs, though the gene of interest targeted by the ASO was in the hepatocytes. More work needs to be done on how the architecture of the liver with differential scavenger receptor expression affects the distribution and efficacy of ASOs.

The Stabilin receptors are only one piece of the endocytosis machinery involved with PS-ASO uptake. Since there is a weak correlation between uptake and efficacy in the GWAS study, it is hypothesized that there are several ASO endocytosis mechanisms and each of these pathways have varying levels of ASO efficacy. Using a screen for several common markers for early and late endocytosis-related proteins, we found that Rab5C, and not Rab5A or Rab5B, is essential for productive uptake of an ASO targeting *malat-1* in 190HARE cells [80]. “Productive uptake” in this context means that the ASO was internalized and efficiently knocked down its target RNA. Another study led by Astrazeneca came to these same conclusions about Rab5C in a screen with several cancer cell lines using an ASO targeting KRAS [81]. Even though the Stabilins were not expressed in these cell lines, Rab5C was a major determinant for productive uptake. It is not yet known if Stabilin-2 and Rab5C interact directly or are just associated with the same early endosomal machinery/vesicles. Clearly, Stabilin-2 is directly involved with tissue accumulation of the PS-ASOs, though how this affects both short- and long-term efficacy of the PS-ASO in the background of multiple mechanisms of internalization still needs to be determined. 

## 6. Receptor Signaling

Although the ecto-domains of Stabilin-1 and Stabilin-2 exhibit high homology, the cytoplasmic domains (consisting of 4% of the receptor) are very diverse and show no homology. To date, few signaling studies have been performed for Stabilin-1 [82], therefore, this section will only focus on the progress made with 190HARE. The investigation into whether the Stab2/190-HARE receptor performs any downstream signaling to modulate cell behavior was to determine if cells are responsive to specific ligands through this receptor. With the use of anti-phosphotyrosine antibodies and confirmed with Pro-Q Diamond (Life Technologies) protein staining, it was determined that the 190HARE and 315HARE (Stabilin-2) receptors are phosphorylated in a time-responsive manner in response to 10 μg of (~250 kDa or 40 nM) HA. For each 190HARE cell line, phospho-ERK1/2, and not JNK or p38 signaling, increased up to 30 min and then rapidly lost the signal by 45 min. The HA response was confirmed by repeating some of the key experiments in cells expressing the 190HARE lacking the Link domain (190-HARE∆Link), suggesting that when HA binds to the receptor, the receptor propagates a signal via the mitogen-activated ERK kinases [33]. Phosphorylation of residues within the cytoplasmic domain was predicted to four amino acids (Ser^2497^, Ser^2537^, Tyr^2519^, and Tyr^2531^) with the NetPhos 2.0 program.

**Figure 5:**
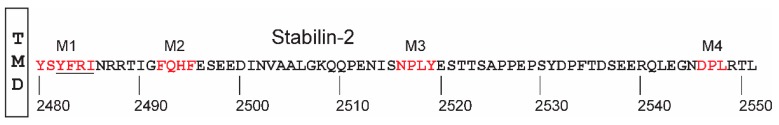
**Cytoplasmic domain of Stabilin-2.** The red lettered domains for Stabilin-2 have been extensively characterized in the 190HARE protein expression system and are known to assist with receptor endocytosis and signaling.

Endocytic receptors often contain one or more specific domains that facilitate entry of the receptor in the forming endosome. The 190HARE cytoplasmic domain contains five endocytic domains with two that significantly overlap, technically forming a single motif adjacent to the transmembrane domain (Figure 5, underlined). Are all four of these domains essential for receptor function in terms of cycling through the endocytic pathway and ligand uptake or is there some redundancy in their functions? Using site-directed mutagenesis, expression levels and HA binding of the mutant 190-HARE receptor did not differ significantly from the WT 190HARE. However, the deletion of the first three domains (M1-M3) reduced endocytosis of HA by about 50% with the mutant lacking Tyr^2519^ the most severely affected. Double- and triple-deletion mutants were made to zero in on the significance of the redundancy of these motifs and a triple mutant lacking M1,M2, and M4 while retaining Tyr^2519^ reduced endocytosis by 42%, while the same triple mutant that was lacking Tyr^2519^ had a reduction of HA uptake of 95% when compared to WT [83]. Interestingly, swapping only the Tyr^2519^ to alanine had only a 5% reduction in endocytosis of HA, suggesting that efficient ligand uptake, receptor routing through the cell, ligand delivery to lysosomes, and recycling of the receptor to the membrane surface is a coordinated effort utilized by all four motifs of 190HARE [83].

Not all ligands interact with Stabilin-2 in the same manner, or in other words, not all ligands induce a signaling response. Stabilin-2 is expressed in areas where there is a multitude of ligands in lymphatic and circulatory fluids. In these settings, both free and membrane-bound glycosaminoglycans and other ligands interact with a variety of receptors that affect cellular behaviors [84]. A subset of Stabilin-2 ligands was tested for nuclear factor (NF-kB) stimulation for the potentiation of gene expression. It was discovered that HA, heparin, dermatan sulfate, and acetylated low-density lipoprotein (acLDL) stimulated the dual-luciferase reporter assay for NF-kB induction, whereas chondroitin sulfates (CS) Types A, C, D, and E did not stimulate luciferase activity [85]. It must be noted here that HA, CS-A, CS-C, CS-D all bind with the Link domain in contrast to heparin and acLDL, which do not interact with the Link domain. Dermatan sulfate and CS-E have higher affinity for the heparin binding domain than the Link domain [67]. Since the Link domain was known to be glycosylated uniquely with sialylated modified glycans [86], we asked if the glycan had an impact on the ligand–receptor interaction in terms of signal propagation. The deletion of Asn^2280^ located within the Link domain and the residue hosting the glycan resulted in the attenuation of both ERK and NF-kB signaling in response to HA stimulation, but not to acLDL, heparin, or dermatan sulfate stimulation [87]. The physical nature of the ligand also induces cellular signaling responses. For example, very large HA over 400 kDa and very small HA under 40 kDa do not activate NF-kB-mediated gene expression even though the receptor will bind with these ligands [85]. The significance of HA size may have to do with physical properties of how the polysaccharide interacts with the protein receptor such as the rod-to-coil transition of HA at about 200 kDa. Likewise, low-molecular-weight heparin does not activate NF-kB unless there is a relatively high concentration in contrast to heparin that induces the NF-kB signal at much lower concentrations [88]. Low-molecular-weight heparin has fewer 3-*O*-sulfated residues to interact with Stabilin-2 than the unfractionated or unprocessed heparin [58,65]. Apparently, Stabilin-2 does not interact with its ligands in an on–off manner, but interactions are on a spectrum of affinities based on the physical and molecular characteristics of the ligand. Signaling responses stimulated by PS-ASOs have yet to be determined. 

Arguably, the most important cytoplasmic domain/motif for both HA and heparin (and similar ligands) and induced activation of both NF-kB and ERK1/2 is the NPLY motif in which the Tyr^2519^ is likely phosphorylated. Endocytosis and propagation of cellular signaling responses that induce changes in cellular behavior are mutually exclusive. As mentioned previously, a deletion mutant of M1, M2, and M4 reduces endocytosis by nearly one-half of the normal rate, however, the ERK1/2 activation required the NPLY motif or M3. This was verified by co-immunoprecipitation of the ERK complexes which were detected on all cytoplasmic mutants except those with a deletion of M3 [88]. With the confirmation that 190HARE is involved with the ERK1/2 and NF-kB signaling and gene induction, this brings us to a hypothesis about the overall role of Stabilin-2 in physiology. Since the ligand chemistry and Stabilin-2/HARE both have multiple parameters for interaction and ligands may bind simultaneously [67], this receptor may act as a sensor for homeostasis in which several inputs (ligands) may elicit responses from the tissues where Stabilin-2 is expressed [88,89]. This would mean that the change in ligand population profiles in lymphatic or hematologic circulation under diseased or injury conditions may induce a counter-response from Stabilin-2-expressing tissues to stabilize the internal environment and attempt to restore homeostasis. Evidence for this lies in the differential signaling activity for HA of different sizes [85]. In addition, the liver SEC which is located at the interface of the liver parenchyma and blood circulation plays an active role in maintaining homeostasis. It is thought that this cell type of the liver promotes an anti-inflammatory and antifibrotic environment during early stages of non-alcohol fatty liver disease [90]. The mechanism for doing this lies in the receptors that are expressed at the cell surface and both Stabilins are robustly expressed in these cells of the liver. 

## 7. Conclusions/Perspectives

The Stabilins are an enigmatic receptor family in which their entire coordinated physiological functions are still not well understood. How does a receptor bind multiple ligands and know how to propagate signal transduction based on the ligand? What is the relationship of the isoforms of Stabilin-2 to each other and with Stabilin-1 when expressed within the same cell? Though there have been multiple splice variants of Stabilin-2 discovered on the transcript level [20], are these proteins translated and expressed by their respective tissues? What is the overall physical structure of each receptor and how does that relate to their respective functions? These and other questions still need to be addressed in order to understand the biochemical and physiological role of each Stabilin receptor.
